# Analysis of the Tensile Deformation Behaviors and Microstructure Characterization under Various Temperatures of MarBN Steel by EBSD

**DOI:** 10.3390/ma16062243

**Published:** 2023-03-10

**Authors:** Tongfei Zou, Meng Liu, Yifan Cai, Quanyi Wang, Yunqing Jiang, Yunru Wang, Yubing Pei, Hong Zhang, Yongjie Liu, Qingyuan Wang

**Affiliations:** 1 Failure Mechanics and Engineering Disaster Prevention Key Laboratory of Sichuan Province, College of Architecture and Environment, Sichuan University, Chengdu 610065, China; 2 Key Laboratory of Deep Underground Science and Engineering, Ministry of Education, Sichuan University, Chengdu 610065, China; 3 State Key Laboratory of Long-Life High-Temperature Materials, Dongfang Turbine Co., Ltd., Deyang 618000, China; 4 School of Architecture and Civil Engineering, Chengdu University, Chengdu 610106, China

**Keywords:** temperature, tensile behavior, EBSD, MarBN steel, microstructure characterization

## Abstract

The uniaxial tensile behavior of MarBN steel with a constant strain rate of 5 × 10^−5^ s^−1^ under various temperatures ranging from room temperature to 630 °C was analyzed. This study aimed to identify the effect of the temperature on the tensile behavior and to understand the microstructure deformation by electron backscatter diffraction. The tensile results showed that the yield and ultimate tensile strength decreased with increasing temperature. Serrated flow was observed from 430 °C to 630 °C. The electron backscatter diffraction analysis showed that the low-angle grain boundaries decreased at the medium deformation and increased at the maximum deformation. In contrast, they decreased with increasing temperatures. In addition, the number of voids increased with the increasing plastic strain. As the strain increased, the voids joined together, and the tiny cracks became larger and failed. Three mechanisms were responsible for the tensile deformation failure at various temperatures: grain rotation, the formation and rearrangement of low angle grain boundaries, and void nucleation and propagation. Finally, the formation of the low-angle grain boundaries and voids under different degrees of deformation is discussed.

## 1. Introduction

In order to reduce CO_2_ emissions, ultra-supercritical (USC) plants should be used to improve power generation efficiency in the 42–46% range, in which the steam temperature is at least 600 °C at 300 bar work pressure [1,2,3], which imposes a high strength requirement on the engineering materials used in this field. For the pressure vessel and piping materials applied in USC projects, the capability to withstand tensile loads in elevated temperatures is vital. MarBN steel has been chosen as a candidate material and designed for USC applications above 600 °C, in which the boron and nitrogen stabilize the martensite laths to improve the mechanical strength in high-temperature low-cycle fatigue (LCF) [4,5,6,7].

The low-cycle fatigue properties of MarBN steel and their tensile behaviors at low and medium temperatures have been reported. Barrett et al. [8,9] presented a dislocation-based constitutive model for high-temperature microstructure degradation to predict the LCF behavior at 400–600 °C. Zhang et al. [10,11] reported that cyclic softening during LCF was related to the laths and grain rotation size and indicated that the material exhibited a non-mashing behavior, and a coarsening microstructure was observed at high strain ratios. Verma et al. [12] observed the serrated flow from 250 to 350 °C at a strain rate of 10^−4^ s^−1^ for modified 9Cr-1Mo steel, which was related to the decohesion of the interface of the carbide particles and the austenite grain boundaries. However, few investigations have focused on the tensile behavior at different temperatures and the microstructure evolution, especially using electron backscatter diffraction (EBSD) for the systematic analysis of MarBN steel. Thus, in this work, the following three issues concerning the tensile behavior are investigated with the microstructure characterization using EBSD: (1) the tensile mechanical behavior of MarBN steel at various elevated temperatures; (2) the evolution of the microstructure at high temperatures; and (3) the analysis of the failure mechanism of the material from its microscopic characteristics.

The current study examined the tensile behavior with a constant strain rate of 5 × 10^−5^ s^−1^ at room temperature (RT), 430 °C, and 630 °C. Then, the different degrees of deformation of the microstructure were observed and analyzed by EBSD. The paper is organized as follows. In Section 2, the material and experimental procedures are described. In Section 3, the tensile behavior at RT, 430 °C, and 630 °C is presented and analyzed. In addition, the deformation of the microstructure at different temperatures observed by EBSD is presented. Then, the relationship between the tensile properties and the microstructure is discussed. Furthermore, the formation of the geometry for necessary dislocations (GNDs) and voids is deduced and analyzed. Finally, the conclusion is presented in Section 4.

## 2. Materials and Methods

**Figure 1:**
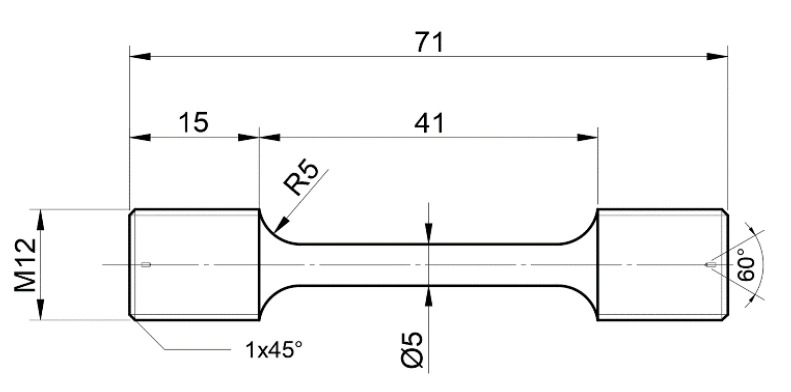
Uniaxial tensile tests specimen (unit: mm) [10].

Martensitic refractory steel (named MarBN steel) was used in the current study. It has a nominal composition (wt. %) of 0.10 C, 9.16 Cr, 0.06 Si, 0.20 Mn, 0.20 Mo, 0.40 Ni, 0.08 Nb, 2.95 W, 2.82 Co, 0.20 V, and Fe as balance [10]. To obtain a stable microstructure, the material was processed with a heat treatment procedure, including solution treatment, i.e., 1250 °C/2 h + 1300 °C/2 h + air cooling (AC), and an aging treatment, i.e., I: 1150 °C/6 h + AC; II: 850 °C/20 h + AC. A uniaxial tensile test (SHIMADZU Corp., Japan) was conducted with a constant strain rate of 5 × 10^−5^ s^−1^ under different temperatures, including RT, 430 °C, and 630 °C. A cylindrical sample with a gauge length of 5 mm in diameter and 41 mm in length [10] was used to analyze the tensile property, as shown in Figure 1. At the same time, the resistance furnace (SHIMADEN Corp., Japan) was used to heat the specimens with the temperature fluctuation at ±5 °C. The test was repeated three times, and the average values were used for each specimen.

EBSD was employed using an FEI Quanta 450F field emission scanning electron microscope (SEM, EDAX Corp., USA) coupled with an Oxford HKL system to obtain the crystallographic information and microstructure. Samples at every temperature, every 1000 μm × 1000 μm in size, were selected along the loading direction. The acceleration voltage of the EBSD was 15 kV, and the scan step size range was 0.05 μm to 0.2 μm. Before measuring, the samples for EBSD observation were prepared as follows: Firstly, a thin sheet of 5 mm in length, 5 mm in width, and 1 mm in thickness was taken by a cutting machine (JUNMA machinery factory, Taizhou, China). Mechanical grinding was carried out using 400#, 1000#, 2000#, and 3000# SiC water-grinding papers. Then, electron polishing was carried out at room temperature. The polishing solution composition was 7% perchloric acid and 83% ethanol, and the current density was 450 mA/cm^2^ for about 1 min. Several statistical analyses were calculated from the EBSD for each sample, including the image quality (IQ), grain boundaries (GBs), grain misorientation, and kernel average misorientation (KAM). The IQ describes the quality of the Kikuchi patterns and evaluates the stored energy or dislocation density [13,14] reflected by the strain distribution. GBs represent the different grain boundaries, such as the low angle grain boundaries (LAGBs, <15°) and the high angle grain boundaries (HAGBs, >15°), which are related to the dislocation distribution and movement. KAM is the critical parameter for evaluating the plastic deformation behavior using local misorientation, indicating the dislocation density [15]. Moreover, before the tensile tests, the specimens characterized by transmission electron microscopy (TEM, Thermo Fisher Scientific Inc., Waltham, MA, USA) with energy-dispersive X-ray spectroscopy (EDS, Thermo Fisher Scientific Inc., USA). In order to determine the fraction of phase in the MarBN steel, X-ray diffraction (XRD, Bruker Corp., Bremen, Germany) tests were performed using a micro-area XRD device, Bruker D8 Focus. Co was used as the target, the wavelength (λ) was set to 0.178897 nm, the voltage was 35 kV, and the current was 40 mA.

## 3. Results and Discussion

### 3.1. Initial Microstructure Characterization

**Figure 2:**
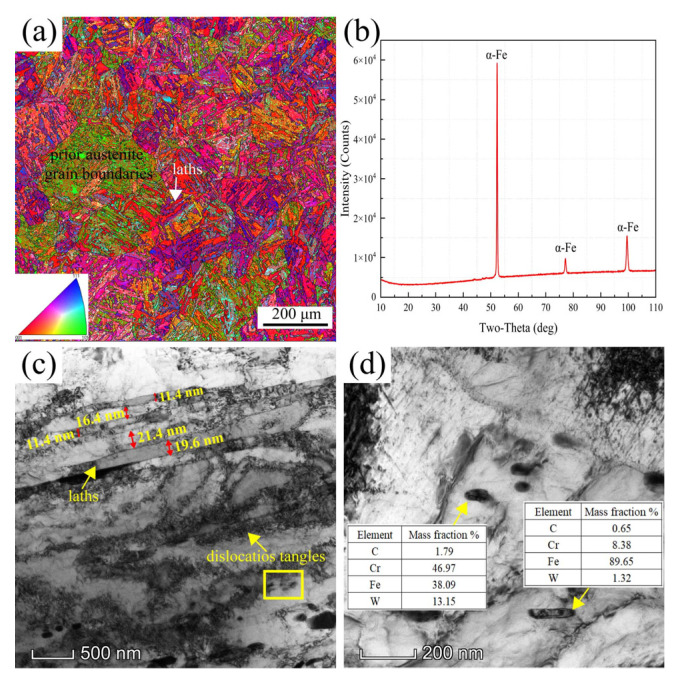
The microstructure of the MarBN steel: (**a**) Inverse polo figures plus GBs; (**b**) image of the XRD analysis results; (**c**) TEM of the microstructure; and (**d**) enlarged view of the yellow rectangle in (**c**).

The microstructure of the MarBN steel before the tests was examined by EBSD and TEM, as shown in Figure 2. Figure 2a is the inverse pole figure (IPF), which contains the prior austenite grain boundaries (PAGBs) and the parallel martensite laths. The average grain size, shown in Figure 2a, was about 13.2 μm based on the statistical results of the Image-Pro software. Moreover, the fractions of HAGBs and LAGBs were approximately 61.31% and 38.69%, respectively. The 100% body-centered cubic (BCC) phase was also observed by XRD analysis performed on the base material, as shown in Figure 2b.

In addition, the TEM microstructures are presented in Figure 2c,d. As seen in Figure 2c, the width of the martensite laths ranged from 11.4 nm to 21.4 nm. It was a nano lath responsible for strengthening the mechanical properties of the material. Furthermore, dislocations entangled along the PAGBs were observed. Precipitations were observed within and along the grain boundaries. Figure 2d is the enlarged view of the yellow rectangle in Figure 2c, where two types of precipitations were analyzed by EDS, the Cr-Fe-rich MX carbides and the Fe-rich M_3_C carbides, respectively.

### 3.2. Tensile Behavior and Temperature Sensitivity

**Figure 3:**
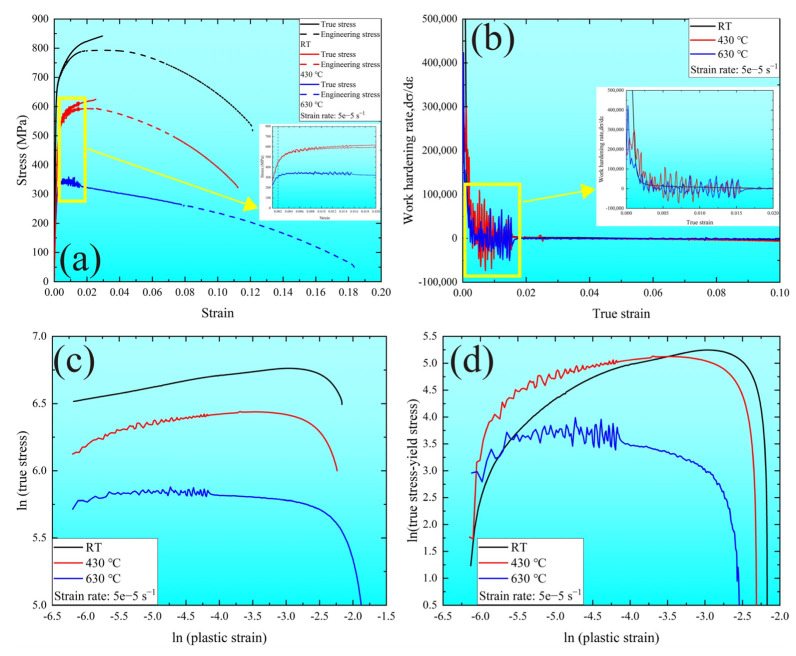
The tensile behavior at a strain rate of 5 × 10^−5^ s^−1^ at RT, 430 °C, and 630 °C: (**a**) The stress–strain curves; (**b**) the work hardening rate curves; and the strain hardening exponent using (**c**) Equation (1) and (**d**) Equation (2).

The stress–strain curves with a constant strain rate, i.e., 5 × 10^−5^ s^−1^ at various temperatures, are presented in Figure 3. The tensile properties, yield ($\sigma_{s}$), and ultimate tensile strength ($\sigma_{ult}$) decreased with the temperature increase. The previous references reported similar results at different strain rates of 5 × 10^−3^ s^−1^ [10]. More details related to the tensile properties of the MarBN steel are presented in Table 1. In addition, to check the hardening capacity at various temperatures, the value of the ratio of ultimate tensile strength to the yield strength minus one was computed, giving 0.28, 0.37, and 0.18 at RT, 430 °C, and 630 °C, respectively. This indicated that softening behavior was apparent at 630 °C. In Figure 3a, three deformation stages are presented along the stress–strain curves at different temperatures, the elastic region fitted by a linear function, the oscillation region between the yield strength and about a 0.02 strain at 430 °C and 630 °C, softening, and finally, the failure region. The work hardening rate curves based on the true stress–true strain curves at various temperatures are presented in Figure 3b, in which the work hardening rate was significantly larger when the strain was below 0.002. Then, the oscillation behavior appeared at 430 °C and 630 °C. However, the rate became constant above the 0.02 strain, and softening and necking occurred. This behavior also depended on the temperature and agreement with the stress–strain curves, as shown in Figure 3a.

Moreover, to understand the strain hardening behavior of the MarBN steel during the plastic deformation region at various temperatures, the strain hardening exponent was used based on the Hollomon [16] and Ludwik [17] relationship as follows:

(1) $$\sigma =k_{1}\varepsilon_{p}^{n_{1}}$$

(2) $$\sigma =\sigma_{y}+k_{2}\varepsilon_{p}^{n_{2}}$$

where $n_{1}$ and $n_{2}$ are the strain hardening exponents; $k_{1}$ and $k_{2}$ are the material parameters; and σ and $\sigma_{y}$ are the true stress and yield strength, respectively. The double logarithmic curves fitted by Equations (1) and (2) are provided in Figure 3c,d, respectively. The slope of the curve in the stable region is defined as the strain hardening exponent, where the curves at 630 °C were the lowest using Equations (1) and (2). However, there was a difference in the curves at RT and 430 °C, resulting from the different fitting equations, as Equation (1) contained both the elastic and plastic deformation, while Equation (2) only had the plastic deformation. The trend of the strain hardening exponent fitted by Equation (1) was similar to the hardening capacity. Furthermore, the strain hardening exponent fitted by Equation (2) had better regularity, as shown in Table 1. Therefore, the strain hardening exponent $n_{2}$ was sensitive to the temperature of MarBN steel.

**Figure 4:**
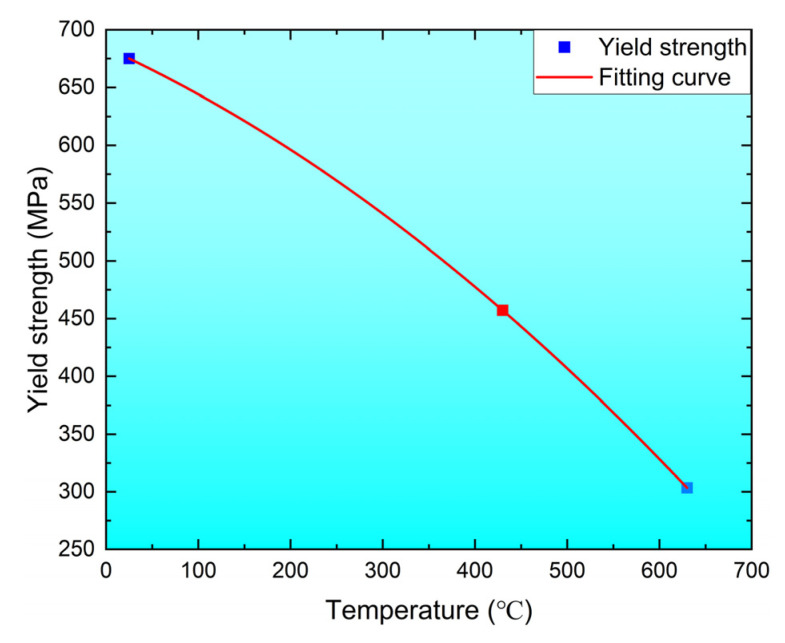
The fitted curve of the yield strength vs. the temperature.

Furthermore, the yield strength vs. temperature is presented in Figure 4, which was fitted by the quadratic polynomial as follows:

(3) $$\sigma_{s}\left(T\right)=684.39-0.36T-3.81\times 10^{-4}T^{2}$$

Generally, the yield strength is related to several contributions, such as the solid solution [18], the grain size [19], and the thermal activation [20], which can be neglected at RT. For the same material and experimental conditions, the yield strength at RT can reflect the solid solution and the grain size contributions. Therefore, coupling Equation (3), the thermally activated contribution is computed as follows:

(4) $$\sigma_{s}^{\mathrm{thermal}\;}\left(T\right)=\sigma_{s}\left(T\right)-\sigma_{s}\left(RT\right)=\left(684.39-0.36T-3.81\times 10^{-4}T^{2}\right)-\sigma_{s}\left(RT\right)$$

where $\sigma_{s}^{\mathrm{thermal}\;}\left(T\right)$ and $\sigma_{s}\left(RT\right)$ are the yield strength of the thermal activation and RT, respectively, and $\sigma_{s}\left(T\right)$ is the yield strength at a defined temperature. As shown in Figure 4, to obtain the yield strength at high temperatures, the thermally activated contribution must be negative, resulting from the disappearance of the low angle boundaries caused by the dislocation annihilation [21] and the decreasing dislocation density at high temperatures [7]. A detailed analysis is presented in the following sections.

Additionally, Figure 3a shows the apparent oscillation behavior between the yield strength and about a 0.02 strain, which was defined as the serrated plastic flow [22,23]. Clearly, there was no serrated flow at RT for the MarBN steel at the tensile strain rate of 5 × 10^−5^ s^−1^. It depended on the temperature from 430 °C to 630 °C at the strain rate of 5 × 10^−5^ s^−1^, wherein the type of serrated flow was the same, indicating that the formation mechanism at both temperatures was the same. In addition, the serrations in the stress–strain curves appeared almost from the beginning of the yield strength and disappeared at about a 0.02 strain before the ultimate tensile strength at both temperatures. Previous references for Cr-based steel [24,25,26,27] observed the dynamic strain aging (DSA) at the temperature range from 225 °C to 420 °C, which was proven by the serrated flow and negative strain hardening index [28,29]. However, in the current investigation, the serrated flow was shown at 430 °C and 630 °C, respectively.

**Figure 5:**
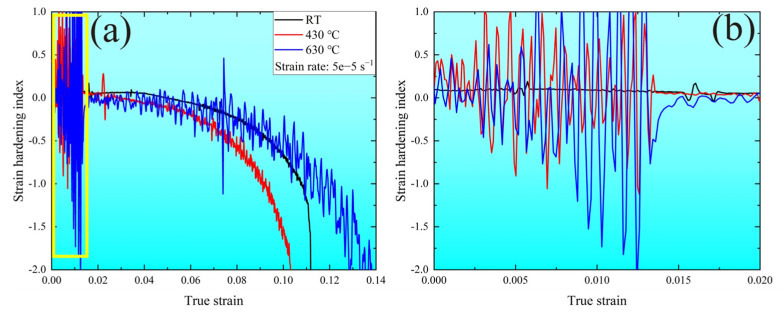
(**a**) The strain hardening index at various temperatures; (**b**) the enlarged image for the yellow rectangle in (**a**).

Figure 5 shows the strain hardening index at various temperatures based on the relationship $\sigma =k\varepsilon^{m}$, where σ and ε are the true stress and the true strain, respectively. As shown in Figure 5a, there was a transparent oscillation region between the yield strength and the 0.02 strain, consisting of the serrated flow region. Above the 0.02 strain, the strain hardening index presented a continuous downward trend at all temperatures. In addition, Figure 5b is the enlarged view for the yellow rectangle in Figure 5a, where the strain hardening index at RT retained a positive constant, while it presented positive and negative trends at 430 °C and 630 °C, respectively. This indicates that the DSA behavior appeared under the serrated flow region at both temperatures, resulting from the interaction between the diffusion of the solute atoms and the gliding dislocation at high temperatures, which led to mobile dislocation and tangling along the slip path [30,31].

In summary, the tensile properties of MarBN steel were temperature dependent, with the yield and tensile strength decreasing at elevated temperatures, and the work hardening rates obtained by the equation fitting showed the same trend. The fitted curve in accordance with the Hollomon equation included the entire tensile stage, i.e., the elastic and plastic deformation, while the fitted equation by Ludwik focused only on the plastic deformation. The appearance of the serrated oscillations also correlated with the effect of temperature, as shown by the dramatic fluctuations of the strain hardening index at high temperatures, while no similar serrations were observed at RT. This is indicative of the DSA behavior at elevated temperatures, where the diffusion of solute atoms was intensified and interacted with the dislocations due to the thermal activation, leading to dislocation entanglements and causing the serrated oscillations.

### 3.3. EBSD Analysis at Various Temperatures

**Figure 6:**
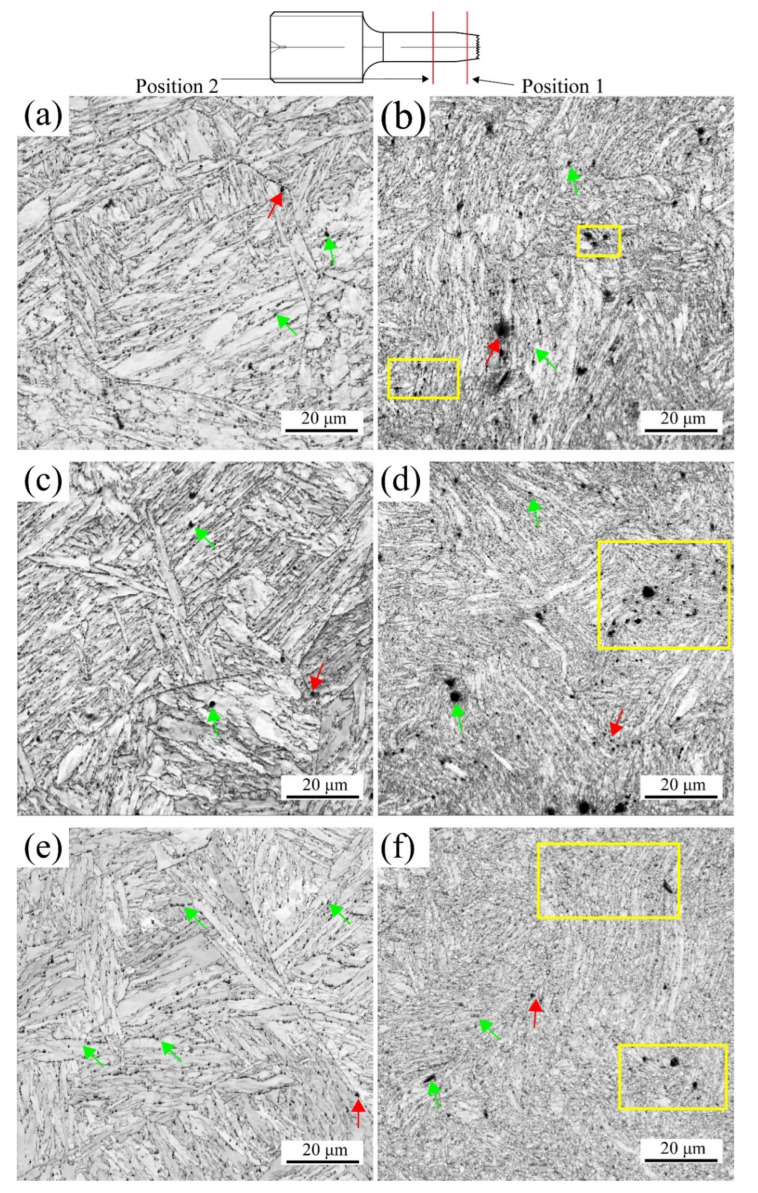
IQ maps for different degrees of deformation at RT (**a**,**b**); 430 °C (**c**,**d**); and 630 °C (**e**,**f**), where the red arrows refer to voids along the PAGBs, and the green arrows represent voids along the boundaries of the laths.

The IQ map presents the Kikuchi patterns related to the lattice distortion [14,32]. Therefore, the void can be described by the black color, which lacks the Kikuchi patterns. Figure 6 shows the IQ maps of the different degrees of deformation (positions 1 and 2) at various temperatures. For the medium deformation (position 2) at different temperatures, as shown in Figure 6a,c,e, the different phases and laths had apparent sharpness and boundaries. The red arrows in Figure 6 represent the voids distributed along the PAGBs, while the green arrows are the voids along the boundaries of the laths, agreeing with previous reports [33,34,35]. Moreover, the number and size of the voids increased and decreased with increased temperatures. At 630 °C, the voids were mainly along the parallel martensite laths. However, at the maximum degree of deformation (position 1), as shown in Figure 6b,d,f, the phases seriously deformed, especially the parallel martensite laths, resulting from the gradient effect on the plastic deformation [36]. This means that the degree of deformation for the martensite laths intensified with the increasing temperatures. In addition, the number and size of the voids increased compared to those at medium deformation, suggesting that the generation and propagation of voids were associated with energy dissipation. Inhomogeneous deformation was observed in different phases. The voids regions were possible at the high KAM and GNDs because of the increased strain. The voids initiated at the martensite laths and PAGBs boundaries, forming the planar arrays of voids that led to the delamination of phases [33]. As the strain increased, the voids were joined together, indicated by the yellow rectangles, which means that the small cracks became the larger ones and eventually failed. Temperature was another crucial factor in improving the atoms diffusion and reinforcing the martensite laths decomposition, leading to the high stress concentration and the martensite lath and PAGBs boundaries.

**Figure 7:**
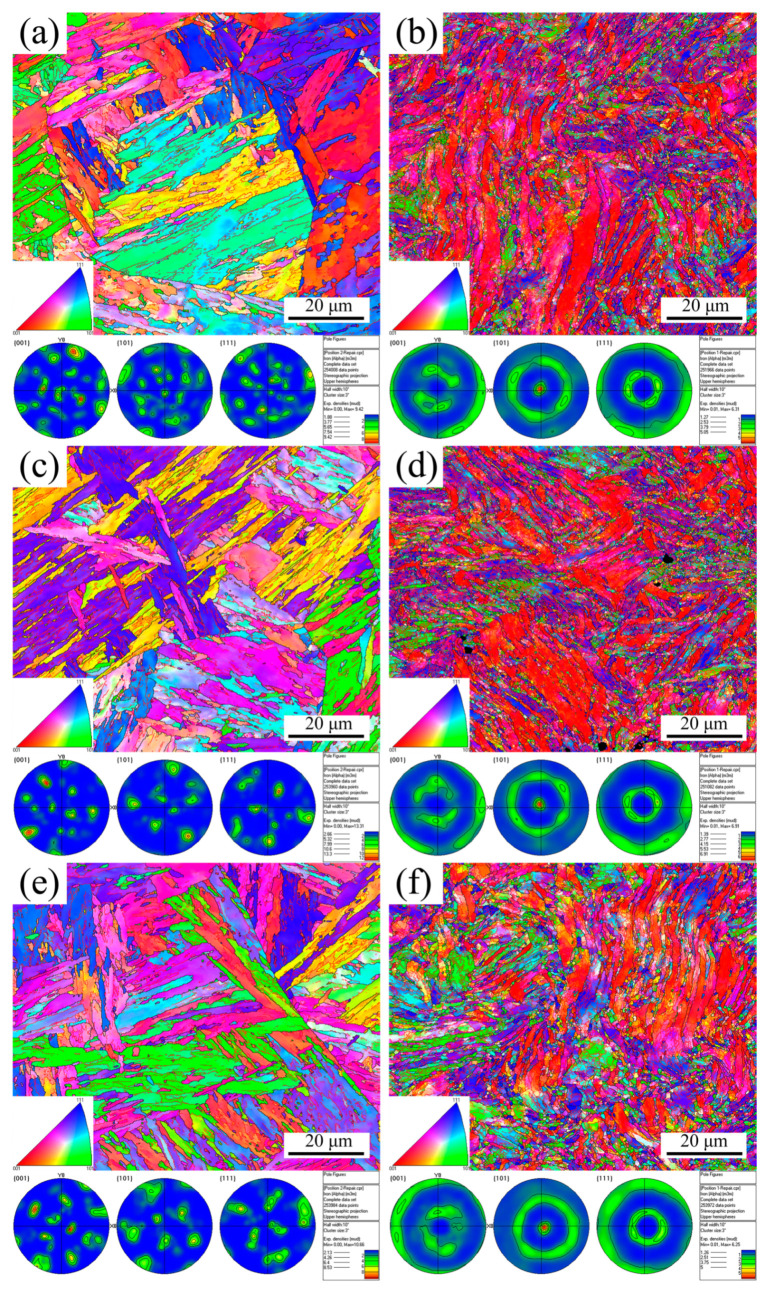
IPF and PF maps for the medium (**a**,**c**,**e**) and maximum (**b**,**d**,**f**) degrees of deformation at RT (**a**,**b**); 430 °C (**c**,**d**); and 630 °C (**e**,**f**).

Figure 7 presents the microstructure according to the IPF and PF maps for the medium and maximum degree deformation at RT, 430 °C, and 630 °C. For the medium deformation illustrated in Figure 7a,c,e, the PAGBs and laths retained a stable geometry. Furthermore, the microstructure had no clear texture at RT. However, the texture was more evident at 430 °C and 630 °C, which means the texture of MarBN steel was related to the temperature, resulting from the decrease in the number of LAGBs, as shown in Table 2. Compared with the initial microstructure presented in Figure 2, the number of LAGBs after the medium deformation dropped significantly, which means the dislocations interacted and annihilated each other during the medium plastic deformation. The diffusion of the solute atoms increased as the temperature increased, which kept up with the movement of the dislocations and reacted with the dislocations to pin them. This explains why the serrated flow appeared at high temperatures during the tensile tests with a constant strain rate. As seen from the maximum deformation presented in Figure 7b,d,f, the PAGBs and laths did not retain a stable geometry, where the PAGBs were divided into several elongated grains due to severe deformation. As the temperature increased, the larger the number of elongated grains, and the greater the tendency to deform. In addition, the apparent texture along the {101} slip plane was observed, which indicated that the grain orientation gradually unified after the maximum plastic deformation at different temperatures. This behavior was independent of the temperatures.

**Figure 8:**
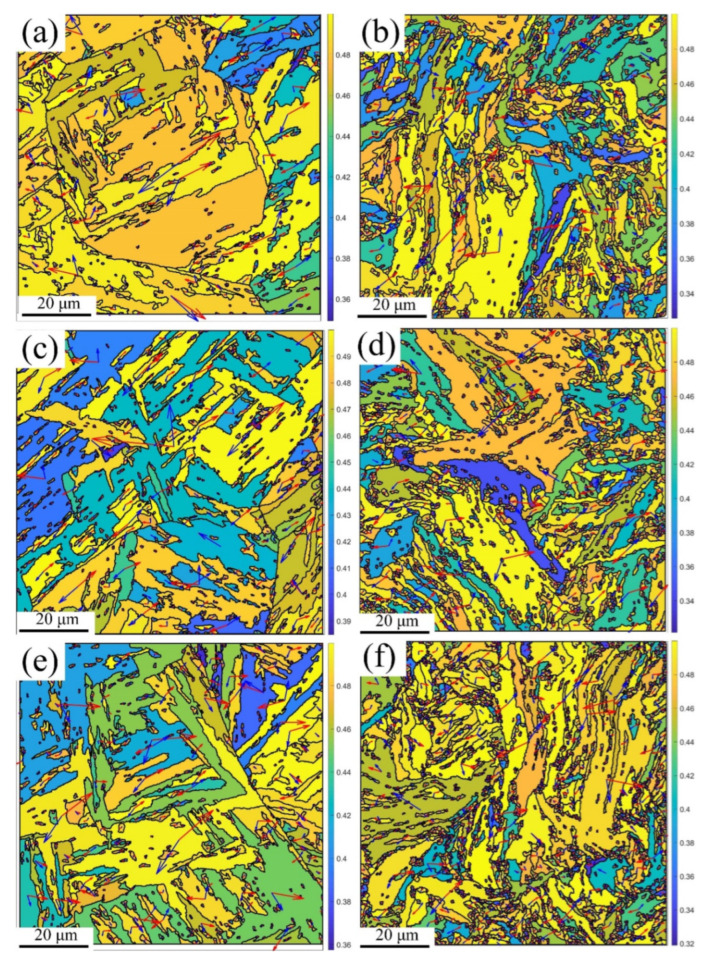
Schmid factor with slip plane (red arrows) and slip direction (blue arrows) maps for the medium (**a**,**c**,**e**) and maximum (**b**,**d**,**f**) degrees of deformation at RT (**a**,**b**); 430 °C (**c**,**d**); and 630 °C (**e**,**f**).

Figure 8 shows the Schmid factor with the slip system at RT, 430 °C, and 630 °C, respectively, using the MTEX [37] toolbox in Matlab, where the small grain size (≤5 μm) was removed to define the slip plane (red arrow) and slip direction (blue arrow). As the Schmid factor at the maximum deformation was larger than that at the medium deformation, the cracks more easily initiated under the maximum deformation. In addition, different slip systems (slip plane and direction) were active in the different phases, such as PAGBs and martensite laths, leading to grain rotation to maintain the deformation continuity under an inhomogeneous microstructure during the plastic strain [38]. It seems that the slip systems increased with the increasing temperatures and strain. Moreover, the grain rotation was responsible for the texture changes [39] under the maximum deformation at various temperatures, as shown in Figure 7b,d,f.

In conclusion, the maps of the IQ, IPF, PF, and Schmid factor were analyzed at various temperatures by means of the EBSD, with samples taken at position 1 near the fracture and position 2 away from the fracture, as shown in Figure 6. The conclusions that can be drawn are as follows: (1) The number of the voids formed inside the material increased with the rise in the temperature, and the size reduced; (2) the increasing temperature led to a reduction in the number of LAGBs, which made the texture more evident; (3) the slip systems within the material increased under the effect of thermal activation; (4) the number of the voids formed at position 1 was greater and larger in size compared to position 2; the geometry of the grains at position 1 was unstable, showing an elongated and twisted morphology, indicating that the grains were rotated; moreover, the Schmid factor at position 1 was also considerably larger. Therefore, the tensile deformation mechanism of MarBN steel is temperature dependent, and the difference in position also influences the magnitude of the deformation.

### 3.4. Formation of the GNDs and Voids

**Figure 9:**
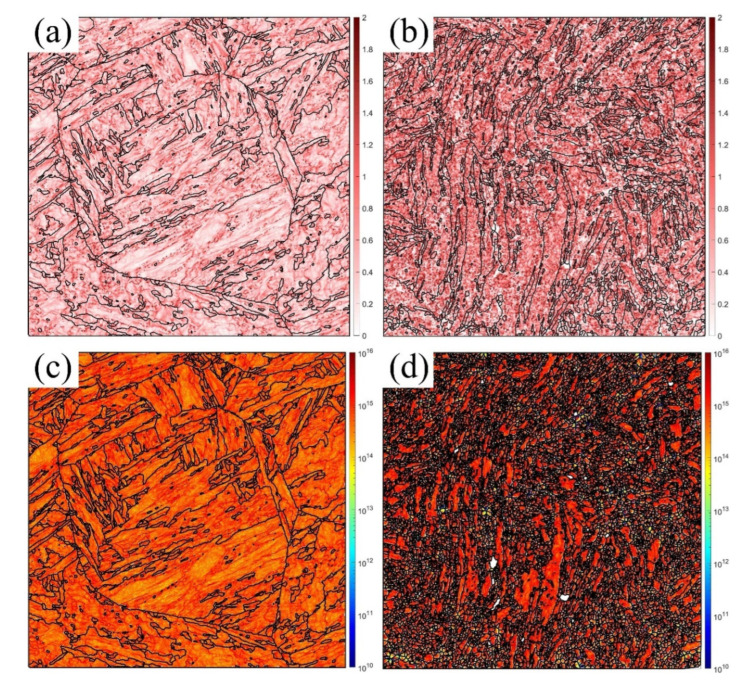
KAM and GND maps for the medium (**a**,**c**), and maximum (**b**,**d**) degrees of deformation at RT.

**Figure 10:**
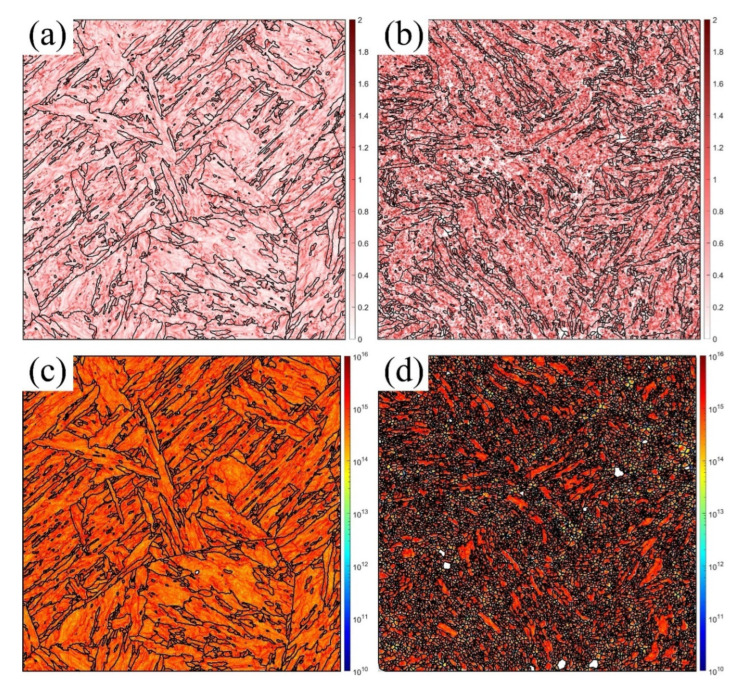
KAM and GND maps for the medium (**a**,**c**) and maximum (**b**,**d**) degrees of deformation at 430 °C.

**Figure 11:**
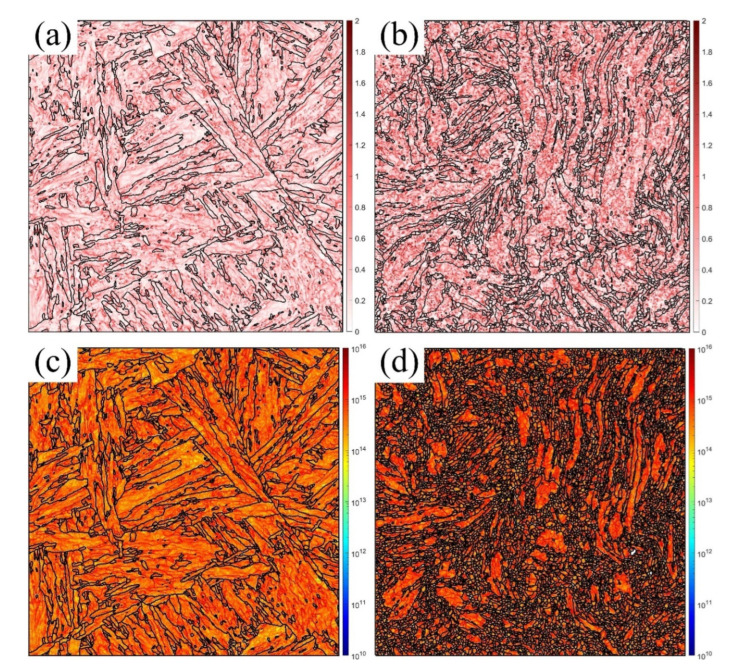
KAM and GND maps for the medium (**a**,**c**) and maximum (**b**,**d**) degrees of deformation at 630 °C.

In addition, the KAM measures the local grain misorientation, which effectively describes the deformation regions [40]. The GNDs are a critical indicator of the geometric continuity in the deformation state [41], resulting from the strain gradient, which rotates the continuum field from point to point [42]. Figure 9, Figure 10 and Figure 11 show the KAM and GND maps under the medium (a,c) and maximum (b,d) degrees of deformation at RT, 430 °C, and 630 °C, respectively. The KAM at the medium deformation was lower than that at the maximum deformation at all temperatures. The GNDs had a similar distribution. The GND density decreased with increasing temperature due to the increased annihilation, which agreed with the previous references [7,10]. The KAM and GNDs at the medium deformation (Figure 9a,c, Figure 10a,c and Figure 11a,c) distributed along the grain boundaries (GBs) and martensite laths due to the inhomogeneous grain structure. This indicates that the GBs were the stress concentration location due to the dislocation pile-up during the plastic strain. Furthermore, as the strain increased, the strain gradient increased and arrived at the maximum before the final failure, as shown in Figure 9b,d, Figure 10b,d, and Figure 11b,d. The KAM increased faster and distributed within all the grains, especially at the elongated PAGBs and laths, which indicated that the grains were inhomogeneously deformed to continue the geometry, and the plastic strain incompatibility of the PAGBs and laths was also shown, resulting from the different soft PAGBs and hard martensite laths [34]. The GNDs had similar rules. However, the GNDs were mostly generated at the small laths and elongated grains, where high LAGBs were observed, as shown in Table 2. Thus, the number of LAGBs was proportional to the GND density [43]. As discussed above, with the increase in the temperature, the percentage of the LAGBs inside the material decreased, and the density of the dislocations reduced due to the intensification of dislocation annihilation at high temperature, resulting in a decrease in the density of GND. Furthermore, by comparing the KAM and GND maps at two different positions, it can be concluded that the KAM and GND at position 2 were mainly distributed at the GBs, where the dislocations stacked up during the deformation and led to stress concentration. However, the KAM and GND at position 1 were distributed along the entire grains, indicating that as the inhomogeneous deformation intensified, the grains began to rotate and distort to maintain the geometric continuity.

As seen in Figure 2, the microstructure of the MarBN steel included soft PAGBs and hard martensite laths. When the uniaxial tensile loading was applied in the different phases, the inhomogeneous deformation for different phases appeared due to the misorientation, resulting in the formation of GNDs along the PAGBs and the GBs of laths, as presented in Figure 9, Figure 10 and Figure 11. As shown in Table 2, compared with an initial number of LAGBs, the LAGBs at the medium deformation decreased, resulting from the dislocation annihilation caused by the dynamic recrystallization behavior (DRX). This can lead to the serrated flow behavior reported by the nickel-based superalloy during compression tests [22]. Therefore, the initial work hardening at the low strain, shown in Figure 3, resulted from a balance between the GNDs and DRX. Temperature played another role in decreasing the dislocation density. However, its effect was weak due to the small gap in the LAGBs at the medium deformation, as shown in Table 2. In addition, the GNDs were rearranged according to the distribution of the LAGBs. Then, a high GND density was formed at the maximum deformation, resulting from the high LAGB density caused by the small and elongated GBs. The dislocations glided across the PAGBs and martensite laths. Then, the GNDs concentrated on the GBs, as shown in Figure 9b,d, Figure 10b,d, and Figure 11b,d. This behavior resulted in more work-hardening before failure, as shown in Figure 3, controlled by the gradient of strain and temperature. With increasing GNDs, the strain gradient was more critical in controlling the KAM and GNDs than the temperature for MarBN steel during the uniaxial tensile process.

**Figure 12:**
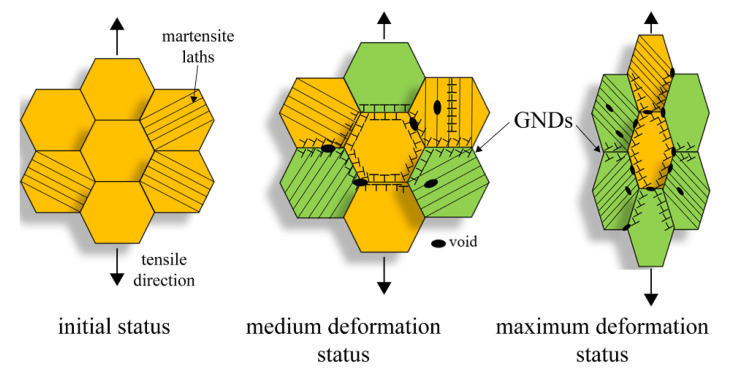
The schematic illustration of the deformation mechanics under the initial, medium, and maximum deformation for MarBN steel.

To sum up, by comparing the results of the EBSD analysis at various temperatures under two different strain gradients, the following conclusions can be drawn: (1) The number and size of the voids formed inside the material increased as the deformation intensified, and the microcracks formed by the interconnection of the voids eventually resulted in failure; (2) in the region near the fracture, the grains were elongated, and the geometry presented a torsional bending morphology, indicating that the degree of deformation was intense; (3) with the elongation of the grains, the percentage of the LAGBs in the maximum deformation region increased, causing the increase in the GNDs in the whole grains, which ultimately generated stress concentration and led to failure. In general, three mechanisms were responsible for the tensile deformation failure at various temperatures, the void nucleation and propagation, the grain rotation, and the formation and rearrangement of the GNDs, as illustrated in Figure 12. Moreover, the temperature effectively decreased the number of the LAGBs and increased the solute atoms diffusion, which further forced the DXR and martensite laths to decompose, resulting in the high stress concentration and the martensite lath and PAGBs boundaries during the tensile deformation.

## 4. Conclusions

The present study investigated the uniaxial tensile deformation behavior of the MarBN steel with a constant strain rate under RT, 430 °C, and 630 °C using EBSD. The temperature dependence on the microstructure and deformation mechanism was observed and studied systematically to obtain the following conclusions:

(1) The tensile behavior was affected by the temperature of the MarBN steel. As the temperature increased, the yield and ultimate tensile strength decreased. The serrated flow was only observed in the temperature range from 430 °C to 630 °C, related to the DSA behavior at high temperatures.
(2) Three mechanisms were responsible for the tensile deformation failure at various temperatures, the grain rotation, the formation and rearrangement of the GNDs, and the void nucleation and propagation.
(3) The KAM and GNDs density had a similar distribution, resulting from the strain incompatibility between different phases. In addition, the temperature played another crucial role in decreasing the number of the LAGBs during the tensile deformation.
(4) The tensile behavior at the medium deformation resulted from a balance between the GNDs and DRX, resulting from the disappearance of the LAGBs caused by the dislocation annihilation. However, the tensile behavior at the maximum deformation was determined by the gradient of strain and temperature. The strain gradient mainly controlled the KAM and GNDs, rather than the temperature in this process.
(5) The number of voids increased with the increasing plastic strain. As the strain increased, the voids were joined together, and the small cracks became larger cracks and finally failed. The temperature was crucial to improving the atoms diffusion, reinforcing the martensite laths’ decomposition, resulting in a high stress concentration along the martensite lath and PAGB boundaries.

## Figures and Tables

**Table 1:** Detailed tensile properties under different temperatures at a strain rate of 5 × 10^−5^.

Strain Rates/s^−1^	Temperature/°C	Yield Strength/MPa	Tensile Strength/MPa	Hardening Capacity	n_1_	n_2_
5 × 10^−5^	RT	675.04	864.73	0.28	0.074	0.432
	430	457.20	626.75	0.37	0.078	0.269
	630	303.52	357.61	0.18	0.004	0.022

**Table 2:** The number of LAGBS and HAGBs under different temperatures at a strain rate of 5 × 10^−5^ s^−1^.

Strain Rates/s^−1^	Temperature/°C	RT-P1 Max-Deformation	430-P1 Max-Deformation	630-P1 Max-Deformation	RT-P2 Medium Deformation	430-P2 Medium Deformation	630-P2 Medium Deformation
5 × 10^−5^	LAGBs	72.03%	68.64%	61.35%	56.39%	54.22%	52.42%
	HAGBs	28.97%	31.36%	38.65%	43.61%	45.78%	47.56%

## Data Availability

The data that support the findings of this work are available from the corresponding author upon reasonable request.

## References

[1] Abe F., Barnard P., Blum R., Chai G., deBarbadillo J.J., Di Gianfrancesco A., Forsberg U., Fukuda M., Hald J., Klöwer J. (2017). Materials for Ultra-Supercritical and Advanced Ultra-Supercritical Power Plants.

[2] Viswanathan R., Henry J., Tanzosh J., Stanko G., Shingledecker J., Vitalis B., Purgert R.U.S. (2013). Program on Materials Technology for Ultra-Supercritical Coal Power Plants. J. Mater. Eng. Perform..

[3] Abe F. (2015). Research and Development of Heat-Resistant Materials for Advanced USC Power Plants with Steam Temperatures of 700 °C and Above. Engineering.

[4] Abe F., Tabuchi M., Semba H., Igarashi M., Yoshizawa M., Komai N., Fujita A. Feasibility of MARBN Steel for Application to Thick Section Boiler Components in USC Power Plant at 650 degrees C. Proceedings of the 5th International Conference on Advances in Materials Technology for Fossil Power Plants.

[5] Abe F. (2008). Precipitate design for creep strengthening of 9% Cr tempered martensitic steel for ultra-supercritical power plants. Sci. Technol. Adv. Mater..

[6] Zhang H., Wang Q., Gong X., Wang T., Pei Y., Zhang W., Liu Y., Wang C., Wang Q. (2021). Comparisons of low cycle fatigue response, damage mechanism, and life prediction of MarBN steel under stress and strain-controlled modes. Int. J. Fatigue.

[7] Wang Q., Wang Q., Gong X., Wang T., Zhang W., Li L., Liu Y., He C., Wang C., Zhang H. (2020). A comparative study of low cycle fatigue behavior and microstructure of Cr-based steel at room and high temperatures. Mater. Des..

[8] Barrett R.A., O’Donoghue P.E., Leen S.B. (2017). A physically-based constitutive model for high temperature microstructural degradation under cyclic deformation. Int. J. Fatigue.

[9] Barrett R.A., O’Donoghue P.E., Leen S.B. (2014). A dislocation-based model for high temperature cyclic viscoplasticity of 9–12 Cr steels. Comp. Mater. Sci..

[10] Zhang X., Wang T., Gong X., Li Q., Liu Y., Wang Q., Zhang H., Wang Q. (2021). Low cycle fatigue properties, damage mechanism, life prediction and microstructure of MarBN steel: Influence of temperature. Int. J. Fatigue.

[11] Gong X., Wang T., Li Q., Liu Y., Zhang H., Zhang W., Wang Q., Wang Q. (2021). Cyclic responses and microstructure sensitivity of Cr-based turbine steel under different strain ratios in low cycle fatigue regime. Mater. Des..

[12] Verma P., Sudhakar Rao G., Chellapandi P., Mahobia G.S., Chattopadhyay K., Santhi Srinivas N.C., Singh V. (2015). Dynamic strain ageing, deformation, and fracture behavior of modified 9 Cr–1 Mo steel. Mater. Sci. Eng. A.

[13] Tarasiuk J., Gerber P., Bacroix B. (2002). Estimation of recrystallized volume fraction from EBSD data. Acta Mater..

[14] Wu J., Wray P.J., Garcia C.I., Hua M., Deardo A.J. (2005). Image Quality Analysis: A New Method of Characterizing Microstructures. Isij Int..

[15] Britton T.B., Birosca S., Preuss M., Wilkinson A.J. (2010). Electron backscatter diffraction study of dislocation content of a macrozone in hot-rolled Ti–6Al–4V alloy. Scr. Mater..

[16] Hollomon J.H. (1945). Tensile deformation. Trans. Am. Inst. Mech. Eng..

[17] Ludwik P., Ludwik P. (1909). Schlußwort (Zusammenfassung). Elemente der Technologischen Mechanik.

[18] Meyers M.A., Chawla K.K. (2009). Mechanical Behavior of Materials.

[19] Gottstein G., Shvindlerman L.S. (2010). Grain Boundary Migration in Metals: Thermodynamics, Kinetics, Applications.

[20] Allain S., Bouaziz O., Chateau J.P. (2010). Thermally activated dislocation dynamics in austenitic FeMnC steels at low homologous temperature. Scr. Mater..

[21] Giroux P.F., Dalle F., Sauzay M., Malaplate J., Fournier B., Gourgues-Lorenzon A.F. (2010). Mechanical and microstructural stability of P92 steel under uniaxial tension at high temperature. Mater. Sci. Eng. A.

[22] Liu X., Fan J., Li K., Song Y., Liu D., Yuan R., Wang J., Tang B., Kou H., Li J. (2021). Serrated flow behavior and microstructure evolution of Inconel 625 superalloy during plane-strain compression with different strain rates. J. Alloys Compd..

[23] Zhang L., Guo P., Wang G., Liu S. (2020). Serrated flow and failure behaviors of a Hadfield steel at various strain rates under extensometer-measured strain control tensile load. J. Mater. Res. Technol..

[24] Chandravathi K.S., Laha K., Parameswaran P., Mathew M.D. (2012). Effect of microstructure on the critical strain to onset of serrated flow in modified 9Cr–1Mo steel. Int. J. Pres. Ves. Pip..

[25] Choudhary B.K. (2013). Influence of strain rate and temperature on serrated flow in 9Cr–1Mo ferritic steel. Mater. Sci. Eng. A.

[26] Lee J., Moon J., Bae J.W., Park J.M., Kwon H., Kato H., Kim H.S. (2021). Temperature- and strain-dependent thermally-activated deformation mechanism of a ferrous medium-entropy alloy. Intermetallics.

[27] Palaparti D.P.R., Choudhary B.K., Isaac Samuel E., Srinivasan V.S., Mathew M.D. (2012). Influence of strain rate and temperature on tensile stress-strain and work hardening behaviour of 9 Cr–1 Mo ferritic steel. Mater. Sci. Eng. A.

[28] Keller C., Margulies M.M., Hadjem-Hamouche Z., Guillot I. (2010). Influence of the temperature on the tensile behaviour of a modified 9 Cr–1 Mo T91 martensitic steel. Mater. Sci. Eng. A.

[29] Roy A.K., Kumar P., Maitra D. (2009). Dynamic strain ageing of P91 grade steels of varied silicon content. Mater. Sci. Eng. A.

[30] Rodriguez P. (1984). Serrated plastic flow. Bull. Mater. Sci..

[31] van den Beukel A. (1975). Theory of the effect of dynamic strain aging on mechanical properties. Phys. Status Solidi.

[32] Mironov S., Sato Y.S., Kokawa H. (2000). Electron Backscatter Diffraction in Materials Science.

[33] Kadkhodapour J., Butz A., Ziaei Rad S. (2011). Mechanisms of void formation during tensile testing in a commercial, dual-phase steel. Acta Mater..

[34] Saeidi N., Ashrafizadeh F., Niroumand B., Barlat F. (2015). EBSD study of micromechanisms involved in high deformation ability of DP steels. Mater. Des..

[35] Saeidi N., Ashrafizadeh F., Niroumand B., Barlat F. (2015). EBSD Study of Damage Mechanisms in a High-Strength Ferrite-Martensite Dual-Phase Steel. J. Mater. Eng. Perform..

[36] Martínez-Pañeda E., Deshpande V.S., Niordson C.F., Fleck N.A. (2019). The role of plastic strain gradients in the crack growth resistance of metals. J. Mech. Phys. Solids.

[37] Bachmann F., Hielscher R., Schaeben H. (2010). Texture Analysis with MTEX—Free and Open Source Software Toolbox. Solid State Phenom..

[38] Field D.P., Trivedi P.B., Wright S.I., Kumar M. (2005). Analysis of local orientation gradients in deformed single crystals. Ultramicroscopy.

[39] Bjerkaas H., Fjeldbo S.K., Roven H.J., Hjelen J., Chiron R., Furu T. (2006). Study of Microstructure and Texture Evolution Using In-Situ EBSD Investigations and SE Imaging in SEM. Mater. Sci. Forum.

[40] Shen R.R., Efsing P. (2018). Overcoming the drawbacks of plastic strain estimation based on KAM. Ultramicroscopy.

[41] Nye J.F. (1953). Some geometrical relations in dislocated crystals. Acta Metall..

[42] Ashby M.F. (1970). The deformation of plastically non-homogeneous materials. Philos. Mag. A J. Theor. Exp. Appl. Phys..

[43] Wright S.I., Nowell M.M., Field D.P. (2011). A Review of Strain Analysis Using Electron Backscatter Diffraction. Microsc. Microanal..

